# Development of a *Pichia pastoris* cell factory for efficient production of germacrene A: a precursor of β-elemene

**DOI:** 10.1186/s40643-023-00657-0

**Published:** 2023-07-12

**Authors:** Jintao Cheng, Yimeng Zuo, Gaofei Liu, Dongfang Li, Jucan Gao, Feng Xiao, Lei Huang, Zhinan Xu, Jiazhang Lian

**Affiliations:** 1 Key Laboratory of Biomass Chemical Engineering of Ministry of Education, College of Chemical and Biological Engineering, Zhejiang University, Hangzhou, 310027 China; 2 ZJU-Hangzhou Global Scientific and Technological Innovation Center, Zhejiang University, Hangzhou, 311215 China; 3 Zhejiang Key Laboratory of Smart Biomaterials, Zhejiang University, Hangzhou, 310027 China

**Keywords:** β-Elemene, Germacrene A, *Pichia pastoris*, Microbial cell factories, Metabolic engineering

## Abstract

β-Elemene, an active ingredient found in medicinal plants like turmeric and zedoary, is a sesquiterpene compound with antitumor activity against various cancers. However, its current mode of production through plant extraction suffers from low efficiency and limited natural resources. Recently, there has been an increased interest in establishing microbial cell factories to produce germacrene A, which can be converted to β-elemene by a one-step reaction in vitro. In this study, we constructed an engineered *Pichia pastoris* cell factory for producing germacrene A. We rerouted the fluxes towards germacrene A biosynthesis through the optimization of the linker sequences between germacrene A synthase (*GAS*) and farnesyl pyrophosphate synthase (*ERG20*), overexpression of important pathway genes (i.e., *IDI1, tHMG1*, and *ACS*), and multi-copy integration of related expression cassettes. In combination with medium optimization and bioprocess engineering, the final titer of germacrene A in a 1 L fermenter reached 1.9 g/L through fed-batch fermentation. This represents the first report on the production of germacrene A in *P. pastoris* and demonstrates its advantage in producing terpenoids and other value-added natural products.

**Graphical Abstract:**

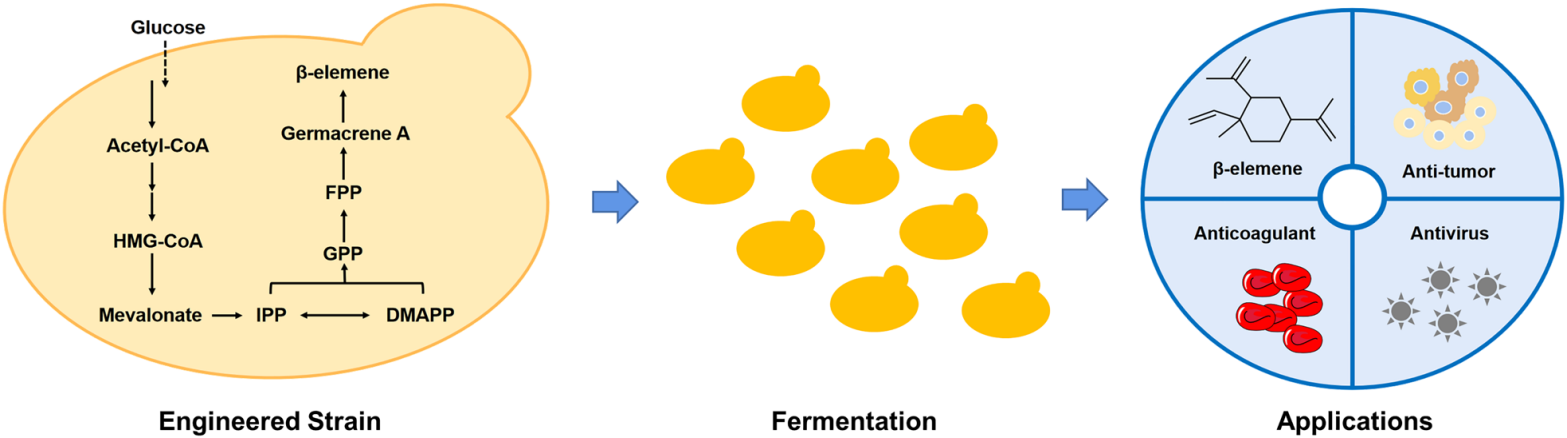

**Supplementary Information:**

The online version contains supplementary material available at 10.1186/s40643-023-00657-0.

## Introduction

Terpenoids represent the largest category of natural products, with significant pharmacological, physiological, and ecological effects (Bohlmann and Keeling 2008). They have a wide range of applications, including medicine, food, and cosmetics. β-Elemene, a sesquiterpene compound derived from Chinese herbal medicine turmeric or zedoary, is a promising anticancer agent with no significant side effects (Chen et al. 2023). At present, β-elemene has demonstrated a broad spectrum of antitumor activity against various cancer types, such as breast cancer, leukemia, prostate cancer, brain cancer, ovarian cancer, cervical cancer, colon cancer, laryngeal cancer, and lung cancer (Lu et al. 2012). While β-elemene is not a direct product of sesquiterpene synthase, it is derived from the precursor germacrene A through intramolecular rearrangement (Hu et al. 2017). Germacrene A can also be converted to β-elemene in vitro (De Kraker et al. 1998). Therefore, biosynthesis of germacrene A using dedicated synthases is a promising approach to produce β-elemene. The traditional method of extracting β-elemene from plants is less efficient and limited in resources (Lu et al. 2012; Sousa et al. 2005). With the rapid development of synthetic biology, microbial cells have attracted significant attention as promising hosts for producing β-elemene.

Germacrene A biosynthesis has been widely studied, and several reports have described its production (Chen et al. 2022; Hu et al. 2017; Broker et al. 2020; Zhang et al. 2021; Li et al. 2022) (Table 1). In microorganisms, germacrene A is mainly produced from farnesyl pyrophosphate (FPP), an intermediate of the mevalonate pathway, catalyzed by germacrene A synthase (GAS). Intramolecular rearrangement of germacrene A under heated or acidic conditions leads to the formation of β-elemene. Hu et al. engineered *Saccharomyces cerevisiae* to produce germacrene A by expressing the lettuce-derived *GAS* (*LTC2*). The titer of germacrene A was further increased to 190.7 mg/L by multi-copy integration of the truncated 3-hydroxy-3-methylglutaryl-CoA reductase (*tHMG1*) expression cassette and fusion expression of *LTC2* and farnesyl pyrophosphate synthase (*ERG20*) (Hu et al. 2017). Subsequently, Zhang et al. also employed *S. cerevisiae* cell factory to optimize the production of germacrene A, via evaluating ten different sources of *GAS*s and optimizing key enzymes and enzyme modification strategies. They achieved a titer of 309.8 mg/L in shake-flask batch culture (Zhang et al. 2021). In addition to *S. cerevisiae*, *Escherichia coli* has also been used as a cell factory for the biosynthesis of germacrene A (Chen et al. 2022; Li et al. 2022).

**Table 1:** Production of germacrene A in metabolically engineered strains

Host	Shake flask fermentation (mg/L)	Fed-batch fermentation	References
*E. coli*	126	–	Chen et al. (2023)
*E. coli*	364	–	Li et al. (2022)
*S. cerevisiae*	190	–	Hu et al. (2017)
*S. cerevisiae*	310	–	Zhang et al. (2021)
*O. polymorpha*	509	4.7 g/L	Ye et al. (2023)
*P. pastoris*	335	1.9 g/L	This study

While *S. cerevisiae* and *E. coli* are commonly used as cell factories for natural product biosynthesis (Wang et al. 2023; Yang et al. 2020), *Pichia pastoris* has gained extensive attention due to its high efficiency in expressing recombinant proteins (Karbalaei et al. 2020). For example, Ma et al. engineered a *P. pastoris* cell factory for efficient expression of human-derived collagen (Ma et al. 2022). *P. pastoris* is also known for its biosafe, well-defined genetic background, metabolic pathways, and regulatory networks, as well as ease of performing high-density fermentation (Liu et al. 2018). With the rapid development of CRISPR/Cas9 genome editing technology and the advantages of recombinant protein expression, *P. pastoris* has been attempted as an efficient cell factory to biosynthesize a variety of natural products, such as catharanthine (Gao et al. 2023), (+)-nootkatone (Wriessnegger et al. 2014), β-carotene (Gao et al. 2022a, b), lycopene (Araya-Garay et al. 2012a, b), xanthophylls (Araya-Garay et al. 2012a, b), α-alkenes (Cai et al. 2022), and α-santalene (Zuo et al. 2022). Notably, Zuo et al. established *P. pastoris* as a cell factory for high-level production of α-santalene, whose titer reached up to 21.5 g/L using fed-batch fermentation (Zuo et al. 2022), representing the highest titer ever reported and indicating its advantage in producing terpenoid natural products. Unfortunately, there have been no reports on germacrene A biosynthesis in *P. pastoris.*

**Fig. 1:**
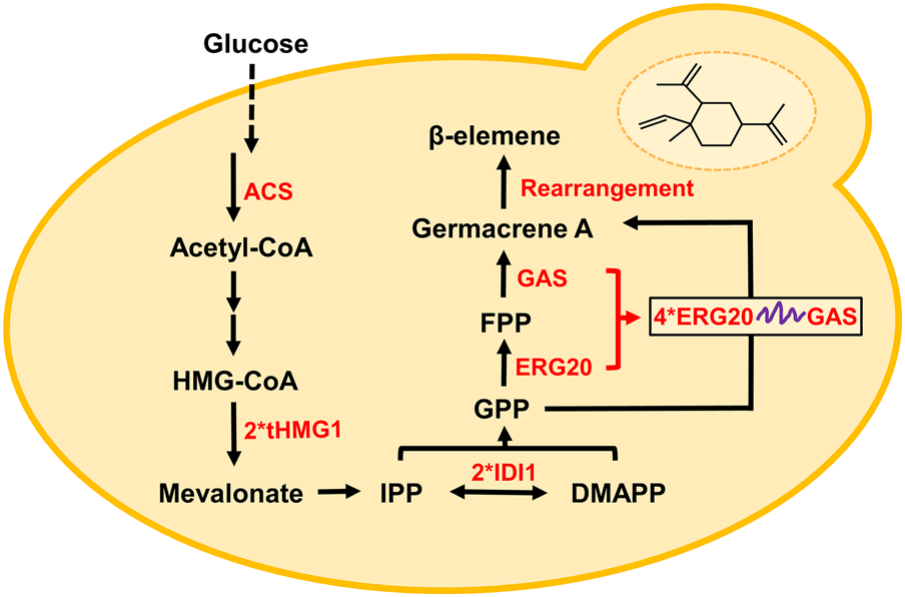
Schematic overview of germacrene A biosynthesis in *P. pastoris.* The overexpressed genes are indicated by red arrows. ACS, acetyl-CoA synthetase; HMG-CoA, 3-hydroxy-3-methylglutaryl-CoA; tHMG1, truncated HMG-CoA reductase; IPP, isopentenyl pyrophosphate; IDI1, isopentenyl diphosphate isomerase 1; DMAPP, dimethylallyl pyrophosphate; GPP, geranyl diphosphate; ERG20, farnesyl diphosphate synthase; FPP, farnesyl diphosphate; GAS, germacrene A synthase

In this study, we aim to construct a *P. pastoris* cell factory for efficient production of germacrene A (Fig. 1). We started by screening various fusion expression linkers between *GAS* and *ERG20*, and found that (PT)4P linker showed the best performance in the biosynthesis of germacrene A. We then regulated copy number of *ERG20-GAS* to increase the titer of germacrene A. Subsequently, we overexpressed the mevalonate pathway genes (*tHMG1* and isopentenyl diphosphate isomerase 1, *IDI1*) and acetyl-CoA synthase gene *ACS* in a multi-copy integration manner to further enhance germacrene A biosynthesis of. Finally, through media optimization and fed-batch fermentation, we achieved the germacrene A titer of 1.9 g/L. When our manuscript was under review, Ye et al. reported the production of germacrene A in *Ogataea polymorpha* with a titer of 4.7 g/L using fed-batch fermentation, by optimizing the mevalonate pathway, enhancing the supply of NADPH and acetyl-CoA, as well as downregulating competing pathways (Ye et al. 2023). Higher production in *O. polymorpha* indicated that NADPH and acetyl-CoA supply as well as competing pathways should be further engineered in *P. pastoris*. Nevertheless, both studies achieved much higher production than that in *S. cerevisiae*, demonstrating the advantage of methylotrophic yeasts in producing terpenoids and other value-added natural products.

## Material and methods

### Strain and plasmid construction

*E. coli* DH5α was used for plasmid construction. *P. pastoris* GS115-Cas9 (GAS0) was used as the parent strain for germacrene A biosynthesis (Gao et al. 2022a, b). The helper plasmids and corresponding sgRNA plasmids were constructed in our previous studies (Gao et al. 2022a, b). The germacrene A synthase gene *GAS* was synthesized by GenScript (Nanjing, China). Key genes in the germacrene A biosynthetic pathway, including *ERG20*, *tHMG1*, *IDI1*, and *ACS*, were amplified from the yeast genome and assembled into the corresponding helper plasmids by digestion ligation or the ClonExpress One-step Cloning Kit (Vazyme Biotech, Nanjing, China). All plasmids, primer sequences, and coding sequences used in this study are listed in Additional file 1: Table S1, Table S2, and Table S3, respectively.

Germacrene A biosynthetic pathway genes were integrated into the *P. pastoris* chromosome using the CRISPR/Cas9 technology (Araya-Garay et al. 2012a, b). Pathway gene expression cassettes were PCR amplified with the homologous arms and co-transformed with the corresponding sgRNA plasmids into the Cas9-expressing strain (Araya-Garay et al. 2012a, b). Electroporation was used for *P. pastoris* transformation (Gao et al. 2022a, b). *P. pastoris* strains constructed in this study are summarized in Table 2 and Additional file 1: Fig. S1. Integration sites for the corresponding pathway gene expression cassettes are listed in Additional file 1: Table S4.

**Table 2:** *P. pastoris* strains constructed in this study

Strain	Genotype	Source
GAS0	*P. pastoris* GS115-*HIS4*::*Cas9*	Gao et al. (2022a, b)
GAS1	GAS0-Int1::*TEF1p*-*GAS*-*AOX1t*	This study
GAS2	GAS0-Int1::*TEF1p-ERG20-AOX1t-GAPp-GAS-CYC1t*	This study
GAS3	GAS0-Int1::*TEF1p-ERG20-GGS-GAS-AOX1t*	This study
GAS4	GAS0-Int1::*TEF1p-ERG20-GGGGS-GAS-AOX1t*	This study
GAS5	GAS0-Int1::*TEF1p-ERG20-(PT)4P-GAS-AOX1t*	This study
GAS6	GAS0-Int1::*TEF1p-ERG20-(PA)5-GAS-AOX1t*	This study
GAS7	GAS5-Int12::*TEF1p-ERG20-(PT)4P-GAS-AOX1t*	This study
GAS8	GAS5-Int32::*TEF1p-ERG20-(PT)4P-GAS-AOX1t-GAPp-ERG20-(PT)4P-GAS-CYC1t*	This study
GAS9	GAS7-Int33::*TEF1p-ERG20-(PT)4P-GAS-AOX1t-GAPp-ERG20-(PT)4P-GAS-CYC1t*	This study
GAS10	GAS8-Int34::*TEF1p-ERG20-(PT)4P-GAS-AOX1t-GAPp-ERG20-(PT)4P-GAS-CYC1t*	This study
GAS11	GAS9-Int20::*TEF1p-IDI1-AOX1t*	This study
GAS12	GAS9-Int20::*TEF1p-tHMG1-AOX1t*	This study
GAS13	GAS9-Int20::*TEF1p-IDI1-AOX1t-GAPp-tHMG1-CYC1t*	This study
GAS14	GAS13-Int21::*TEF1p-IDI1-AOX1t-GAPp-tHMG1-CYC1t*	This study
GAS15	GAS14-Int31::*TEF1p-IDI1-AOX1t-GAPp-tHMG1-CYC1t*	This study
GAS16	GAS14-Int11::*TEF1p-ACS-AOX1t*	This study
GAS17	GAS16-Int16::*TEF1p-ACS-AOX1t*	This study
GAS18	GAS17-Int6::*TEF1p-ACS-AOX1t*	This study

### Media and growth conditions

*E. coli* DH5α was cultured in Lysogeny broth (LB, 5 g/L yeast extract, 10 g/L tryptone, and 10 g/L NaCl). *P. pastoris* was routinely cultured in YPD (10 g/L yeast extract, 20 g/L peptone, and 20 g/L glucose). Bleomycin (100 mg/L), hygromycin (100 mg/L), and geneticin (200 mg/L) were used for the selection of recombinant yeast strains. For germacrene A production, a single colony was picked and inoculated into 5 mL YPD medium and incubated at 30 °C and 220 rpm for 12 h. The culture was then transferred to a 250-mL shake flask containing 50 mL YPD medium with 1% inoculum. After incubation at for 30 °C for 24 h, 5 mL *n*-dodecane was added and fermentation was continued for another 72 h. For media optimization experiments, three different peptones, P (Peptone, pepsin digest of blood fiber-based proteins, Sangon Biotech A505247), P1 (Bacto™ peptone, enzymatic digest of bovine and porcine animal proteins, ThermoFisher BD211677), and P2 (Peptone A, Meat peptone from Bovine, Sangon Biotech A610213), with their differences detailed in Additional file 1: Table S5) and two different yeast extracts, Y (Yeast extract, Sangon Biotech A515245) and Y1 (Bacto™ yeast extract. ThermoFisher BD212750), Additional file 1: Table S5) were tested in different combinations.

### Analytical methods

Cell density (OD_600_) was measured using a UV-2802 spectrophotometer (Lonico Instrument, Shanghai, China). Glucose and ethanol were quantified using previously established methods (Zuo et al. 2022). For the analysis of germacrene A, 1 mL of fermentation broth was centrifuged at 15,000 rpm for 10 min, and the top organic layer was collected. The sample was diluted 100–10,000-fold with ethyl acetate, filtered through a 0.22 μm nylon membrane, and then analyzed using gas chromatography–mass spectrometry (GC–MS). Chromatographic separation conditions: the initial column temperature was set to 50 °C and held for 1 min, followed by an increase to 200 °C at a rate of 10 °C/min, then an increase to 280 °C at a rate of 20 °C/min, and finally held for 3 min. Mass spectrometry conditions: the inlet temperature was set to 280 °C, the flow rate was set to 1.2 mL/min, the ion source temperature was set to 280 °C, the ionization mode was electron ionization (60 eV), the split injection mode was used, the sample injection volume was set to 1 μL, and the quantification was performed using selective reaction monitoring.

### Fed-batch fermentation

A single colony was picked from a fresh agar plate, inoculated into 5 mL medium-filled tube, and incubated for 12 h. The culture was then transferred to a 50 mL of YP2D medium at 1% inoculum, cultured at 30 °C and 220 rpm for 12 h, and inoculated into a 1-L fermenter containing 600 mL YP2D medium. The fermenter was operated at 30 °C and a speed range of 200–600 rpm, with pH maintained at around 5.5. The corrected dissolved oxygen DO was 100%. After 1 day of fermentation, 10% (v/v) *n*-dodecane was added to the culture. Feeding was started when glucose concentration in the fermenter dropped below 1 g/L. The feeding medium contained 10 g/L yeast extract and 400 g/L glucose. During the final feeding process, glucose concentration was maintained at around 1 g/L. Fermentation was performed for 96 h.

## Results

### Construction of a recombinant P. pastoris strain for producing germacrene A

**Fig. 2:**
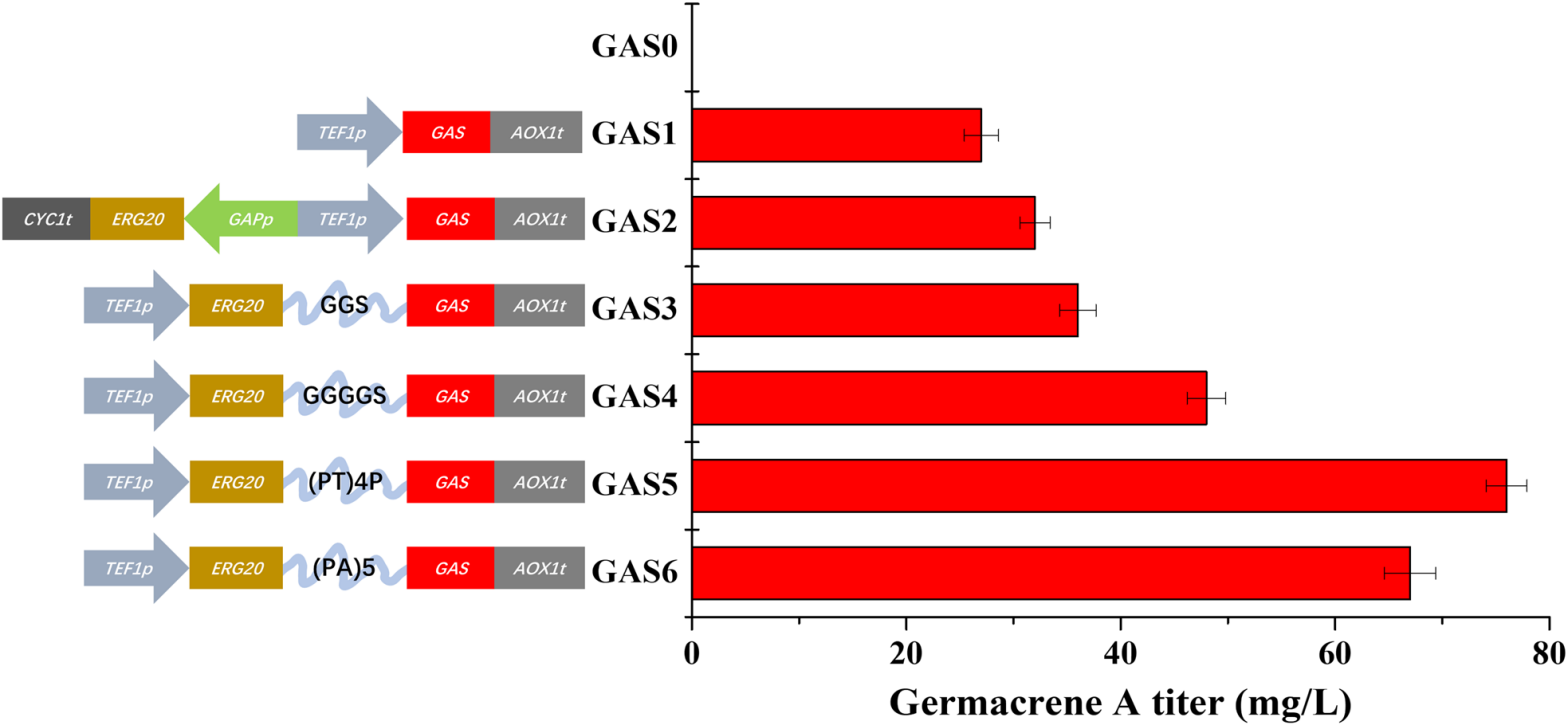
Construction of recombinant *P. pastoris* strains for producing germacrene A. Recombinant strains with different linkers (ERG20-linkers-GAS) were constructed and their titers of germacrene A after fermentation were compared. The fermentation products were detected by GC–MS and the data represent average ± standard deviations of three biological replicates

To construct a recombinant *P. pastoris* strain capable of producing germacrene A, we introduced *Anabaena variabilis*-derived germacrene A synthase gene (*GAS*, which was codon optimized for yeast expression) (Zhang et al. 2021) into *P. pastoris* GS115-Cas9 (GAS0). The resultant strain GAS1, containing a copy of *GAS* expression cassette *TEF1p-GAS-AOX1t*, generated a new peak on GC chromatogram (Additional file 1: Fig. S2), which was further confirmed to be β-elemene by MS analysis (Additional file 1: Fig. S3). The titer of germacrene A produced by GAS1 was determined to be around 27.3 mg/L (Fig. 2), indicating the necessity of further metabolic engineering efforts.

### Fusion expression of GAS and ERG20 for enhanced germacrene A biosynthesis

To increase the titer of germacrene A, we attempted to fuse ERG20 with GAS. Previous studies indicated that fusion enzymes were beneficial for product biosynthesis and the linker sequences between fusion proteins could affect pathway performance (Hu et al. 2017). Thus, we experimented with four different linkers, including GGS, GGGGS, (PT)4P, and (PA)5, leading to the construction of four fusion configurations: ERG20-GGS-GAS, ERG20-GGGGS-GAS, ERG20-(PT)4P-GAS and ERG20-(PA)5-GAS. While GGS and GGGGS are flexible linkers, (PT)4P and (PA)5 represent rigid linkers. Each of these configurations was placed under the control of a strong promoter *TEF1p* (Fig. 2). In addition, we constructed a control strain GAS2, which overexpressed *ERG20* and *GAS* individually.

As shown in Fig. 2, the titer of germacrene A in the recombinant strain GAS2 reached 32.5 mg/L, slightly higher than that of the starting strain GAS1. However, the four fusion-protein expressing strains (GAS3, GAS4, GAS5, and GAS6) exhibited higher titer of germacrene A, indicating that fusion expression could significantly increase the titer of germacrene A. Notably, the fusion-protein (ERG20-(PT)4P-GAS) expressing strain (GAS5) achieved the highest titer of 76.6 mg/L, which was 2.4-fold higher than that of GAS2 (Fig. 2).

**Fig. 3:**
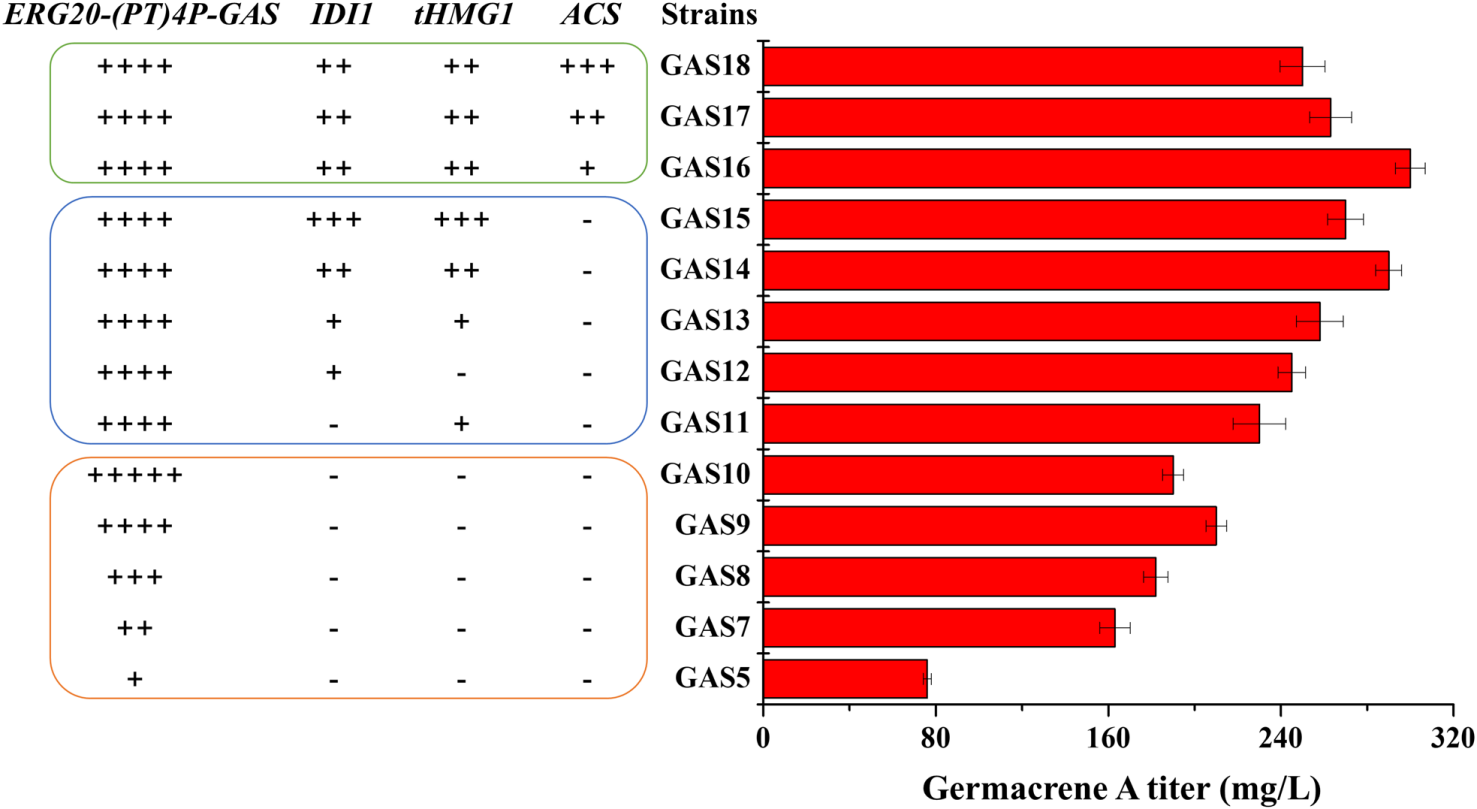
Overexpression and multi-copy integration of the mevalonate pathway genes for enhanced germacrene A biosynthesis. The increase in the copy number of *TEF1p-ERG20-(PT)4P-GAS-AOX1t* enhanced metabolic fluxes towards germacrene A (orange module). Overexpression and multi-copy integration of *IDI1* and *tHMG1* of the MVA pathway increased the titer of germacrene A (blue module). Overexpression of *ACS* increased the supply of acetyl-CoA and accordingly the titer of germacrene A (green module). "+" indicates pathway gene overexpression, and the corresponding number indicates the copy number of the pathway gene expression cassettes. The data represent average ± standard deviations of three biological replicates

To further increase germacrene A production, we employed a multi-copy integration strategy to optimize the copy number of the fusion protein (*ERG20-(PT)4P-GAS*). Our results showed that the titer of germacrene A was increased with more copy numbers of the fusion protein. When the copy number of the fusion protein was four, germacrene A titer of the recombinant strain GAS9 reached 210.0 mg/L (Fig. 3), representing a 7.7-fold increase over the initial strain GAS1.

### Engineering of the MVA pathway to improve germacrene A biosynthesis

FPP is an essential precursor for sesquiterpene biosynthesis in yeast via the mevalonate (MVA) pathway (Zuo et al. 2022). To increase sesquiterpene titer, FPP supply should be enhanced. Common strategies for increasing FPP supply include overexpression of the rate-limiting enzyme encoding genes of the MVA pathway, such as *tHMG1* or isopentenyl diphosphate isomerase (*IDI1*). Thus, we integrated *tHMG1* and *IDI1* expression cassettes individually or in combination into *P. pastoris* GAS9 to construct recombinant strains GAS11, GAS12, and GAS13, respectively. The titer of germacrene A was further increased to 258.4 mg/L in the recombinant strain GAS13 (Fig. 3). These results demonstrated that overexpression of rate-limiting enzymes could increase the metabolic flux of FPP and accordingly the titer of germacrene A.

To further increase the titer of germacrene A, we added an additional copy of the expression cassette (co-expressing *tHMG1* and *IDI1*) to the recombinant strain GAS13. The resultant strain GAS14 was able to produce 291.4 mg/L germacrene A (Fig. 3), an 11.7-fold increase from the starting strain GAS1. However, increasing the copy number of the expression cassette (co-expressing *tHMG1* and *IDI1*) to three failed to further enhance germacrene A production in the recombinant strain GAS15.

### Acetyl-CoA supply engineering to enhance germacrene A biosynthesis

Acetyl-CoA is a direct precursor of the mevalonate pathway, and overexpression of acetyl-CoA synthetase (*ACS*) can increase precursor supply and thus titer of the downstream products (Zuo et al. 2022). To enhance acetyl-CoA supply and further increase germacrene A titer, we introduced the *Salmonella enterocolitica* derived acetyl-CoA synthetase gene *ACS* into the GAS14 strain to obtain the GAS16 strain, which produced 302.6 mg/L of germacrene A (Fig. 3). While the overexpression of *ACS* could further increase the titer of germacrene A, the effect was not substantial. We subsequently added one or two additional copies of *ACS* to GAS16, resulting in the construction of GAS17 and GAS18, respectively. However, germacrene A production was even slightly decreased in GAS17 and GAS18 (Fig. 3), probably due to the metabolic burdens of multi-copy integration of *ACS*. These results indicated that the availability of acetate was limited or the supply of acetyl-CoA was not rate-limiting for germacrene A biosynthesis in *P. pastoris*.

### Media optimization to improve titer of germacrene A in P. pastoris

**Fig. 4:**
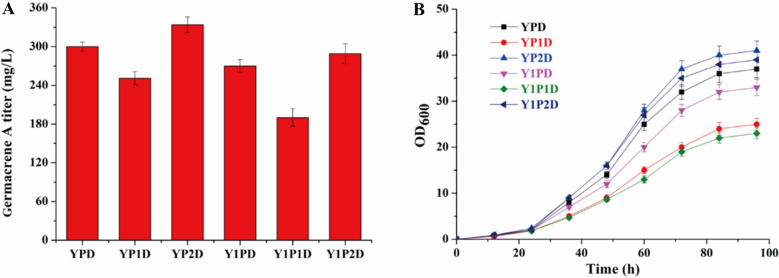
Media optimization for improved germacrene A production in *P. pastoris.* Effects of three different sources of peptone and two different sources of yeast extracts on germacrene A titer (**a**) and cell growth (**b**). The data represent average ± standard deviations of three biological replicates

Media composition is known to have a significant impact on microbial cell growth and metabolite biosynthesis. For example, Zuo et al. successfully increased the titer of α-santalene 1.4-fold by optimizing the nitrogen source in YPD medium (Zuo et al. 2022). Therefore, we evaluated the effects of three different peptones and two different yeast extracts on germacrene A biosynthesis using the best strain GAS16 (Additional file 1: Table S5). Among the combinations tested, YP2D exhibited the most pronounced increase in germacrene A titer compared to the control (YPD), while the titer of germacrene A was reduced in other combinations (Fig. 4a). Specifically, the titer of germacrene A in YP2D was the highest, reaching 334.6 mg/L, 1.12-fold higher than that in YPD. Moreover, as shown in Fig. 4b, the increase in titer was possibly related to better cell growth, further highlighting the effectiveness of media optimization as a strategy to enhance biosynthesis.

### High-level germacrene A production using fed-batch fermentation

**Fig. 5:**
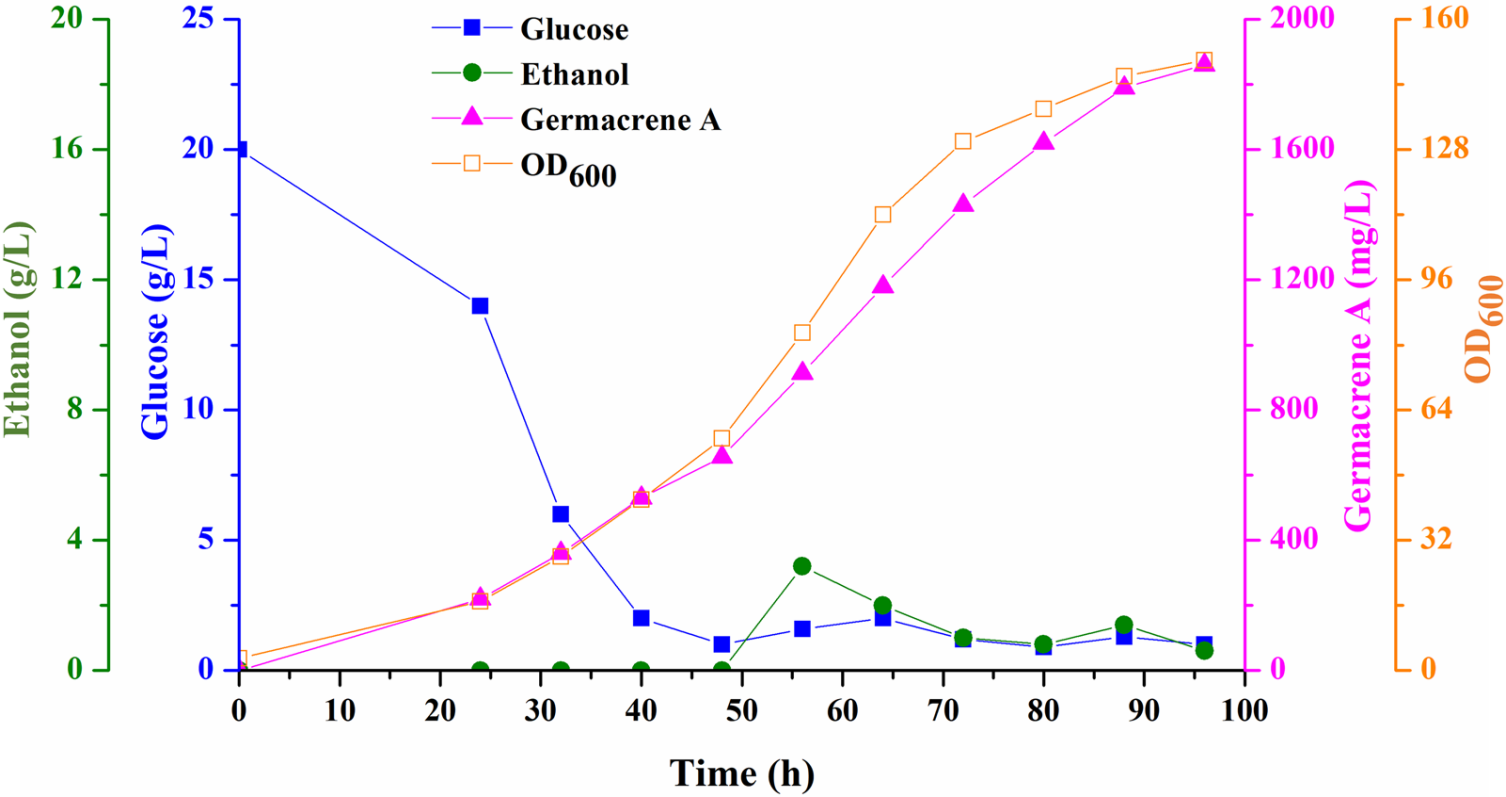
Fed-batch fermentation of GAS16 for high-level production of germacrene A. Fed-batch fermentation was performed in a 1-L bioreactor. Samples were taken every 8 h to analyze the concentration of glucose, ethanol, and germacrene A as well as cell densities

Finally, we performed fed-batch fermentation experiments using the best strain GAS16 in a 1-L bioreactor. By using the fed-batch strategy, the titer of germacrene A reached 1.9 g/L (Fig. 5), which was 6.5-fold higher than that at shake flask level. This was the first report on the production of germacrene A in *P. pastoris*. The experimental results showed that, due to the rapid growth of cells in the late stage of fermentation, a significant amount of glucose was consumed, and the cell density (OD_600_) reached 150.3 after 96 h fermentation. In addition, ethanol content was maintained at a low level. In further experiments, we should consider further optimization of fermentation conditions and feeding processes to improve the titer of germacrene A.

## Discussion

Terpenoids are a diverse class of natural products containing approximately 40,000 known compounds, many of which possess favorable activities and are widely used in various fields such as medicine, food, and cosmetics (Hu et al. 2017; Bohlmann and Keeling 2008). However, traditional methods of plant extraction are time-consuming, labor-intensive, and limited by plant growth (Lu et al. 2012; Sousa et al. 2005; Kato et al. 2021). In recent years, with the rapid development of synthetic biology, efficient production of high-value terpene products using microbial cell factories has attracted increasing attention. Here, we report for the first time on the construction of a *P. pastoris* cell factory for efficient production of germacrene A.

To enhance the production of germacrene A, we employed the strategy of fusing ERG20 and GAS, and tested several fusion linkers including GGS, GGGGS, (PT)4P, and (PA)5. The results showed that ERG20-(PT)4P-GAS (strain GAS5) exhibited the highest titer of germacrene A. Notably, the production of germacrene A in these fusion expression strains (ERG20-GGS-GAS, ERG20-GGGGS-GAS, ERG20-(PT)4P-GAS and ERG20-(PA)5-GAS) was higher than that of the unfused strain GAS2 (ERG20 and GAS overexpressed individually), indicating that fusion expression had a significant impact on enzyme activity. We propose that fusion expression may reduce the loss of intermediate products by shortening the distance between the two enzymes (Additional file 1: Fig. S4), thus enhancing the catalytic activity of the enzyme. Moreover, different linkers were found to affect the biosynthesis performance of the fusion enzymes.

Increasing *GAS* copy number is the most direct strategy to enhance the production of germacrene A, due to higher expression and abundance of the heterologous proteins. Gao et al. employed a similar strategy to increase the production of catharanthine in *P. pastoris* (Gao et al. 2022a, b). In addition, we found that increasing the copy number of *tHMG1* and *IDI1* of the metabolic pathway also significantly enhanced the biosynthesis of germacrene A, due to redirected metabolic fluxes towards the mevalonate pathway and thus the production of germacrene A. Similar genetic manipulations have been reported for the production of α-santalene *in P. pastoris* (Zuo et al. 2022). It is worth noting that only a limited number of genes in the mevalonate pathway, namely *tHMG1* and *IDI1*, were tested in this study, while overexpression and combination of other genes in the mevalonate pathway have not been optimized yet. Combinatorial optimization of these genes has the potential to further enhance the titer of germacrene A. Moreover, the removal of some competing pathways has been reported in *S. cerevisiae* to enhance metabolic fluxes towards the target product (Zhang et al. 2021). Meanwhile, we found that medium composition affected the titer of germacrene A, which was consistent with previous reports (Zuo et al. 2022), indicating that media optimization was also an important strategy to enhance metabolite biosynthesis.

## Conclusions

In summary, we have constructed, for the first time, a *P. pastoris* cell factory for efficient production of germacrene A. Through metabolic engineering and fermentation process optimization, we achieved a titer of 1.9 g/L. This study demonstrates the great potential of *P. pastoris* as a cell factory to produce other high-value terpenoids and natural products.

### Supplementary Information

**Additional file 1: Fig. S1**. Construction procedures of the engineered P. pastoris strains. **Fig. S2**. GC–MS analysis of the production of β-elemene. **Fig. S3**. MS spectrum of β-elemene. **Fig. S4**. Comparison of spatial distances of two proteinsusing different linkers. **Table S1**. A list of plasmids constructed in this study. **Table S2**. A list of primers used in this study. **Table S3**. A list of gene coding sequences used in this study. **Table S4**. A list of integration sites used in this study. **Table S5**. A list of yeast extract and peptone used in this study.

## Data Availability

All data produced or analyzed and materials for this study are available in this article and its additional information flies.

## Abbreviations

ACS: Acetyl-CoA synthetase
HMG-CoA: 3-Hydroxy-3-methylglutaryl-CoA
tHMG1: Truncated HMG-CoA reductase
IPP: Isopentenyl pyrophosphate
IDI1: Isopentenyl diphosphate isomerase 1
DMAPP: Dimethylallyl pyrophosphate
GPP: Geranyl diphosphate
ERG20: Farnesyl diphosphate synthase
FPP: Farnesyl diphosphate
GAS: Germacrene A synthase
