# Supplementary material for: Development of a Pichia pastoris cell factory for efficient production of germacrene A: a precursor of β-elemene
Source: Bioresour Bioprocess. 2023 Jul 12;10(1):38. doi: 10.1186/s40643-023-00657-0 (PMC10992381; doi:10.1186/s40643-023-00657-0)
Supplement: Supplementary file 1 — Additional file 1: Fig. S1. Construction procedures of the engineered P. pastoris strains. Fig. S2. GC–MS analysis of the production of β-elemene. Fig. S3. MS spectrum of β-elemene. Fig. S4. Comparison of spatial distances of two proteinsusing different linkers. Table S1. A list of plasmids constructed in this study. Table S2. A list of primers used in this study. Table S3. A list of gene coding sequences used in this study. Table S4. A list of integration sites used in this study. Table S5. A list of yeast extract and peptone used in this study. [file 40643_2023_657_MOESM1_ESM.docx]

**Supporting Information**

**Development of a *Pichia pastoris* cell factory for efficient production of germacrene A, a precursor of β-elemene**

Jintao Cheng^1,2#^, Yimeng Zuo^1,2#^, Gaofei Liu^1,2^, Dongfang Li^2^, Jucan Gao^1,2^, Feng Xiao^1,2^, Lei Huang^1,2*^, Zhinan Xu^1^, and Jiazhang Lian^1,2,3^*

^1^ Key Laboratory of Biomass Chemical Engineering of Ministry of Education, College of Chemical and Biological Engineering, Zhejiang University, Hangzhou 310027, China

^2^ ZJU-Hangzhou Global Scientific and Technological Innovation Center, Zhejiang University, Hangzhou 311215, China

^3^ Zhejiang Key Laboratory of Smart Biomaterials, Zhejiang University, Hangzhou 310027, China

^#^These authors contributed equally to this work.

*Corresponding authors:

Prof. Lei Huang (Email: lhuangblue@zju.edu.cn)

Prof. Jiazhang Lian (E-mail: jzlian@zju.edu.cn)

Key Laboratory of Biomass Chemical Engineering of Ministry of Education, College of Chemical and Biological Engineering, Zhejiang University, Hangzhou 310027, China

**Supplementary Figures**


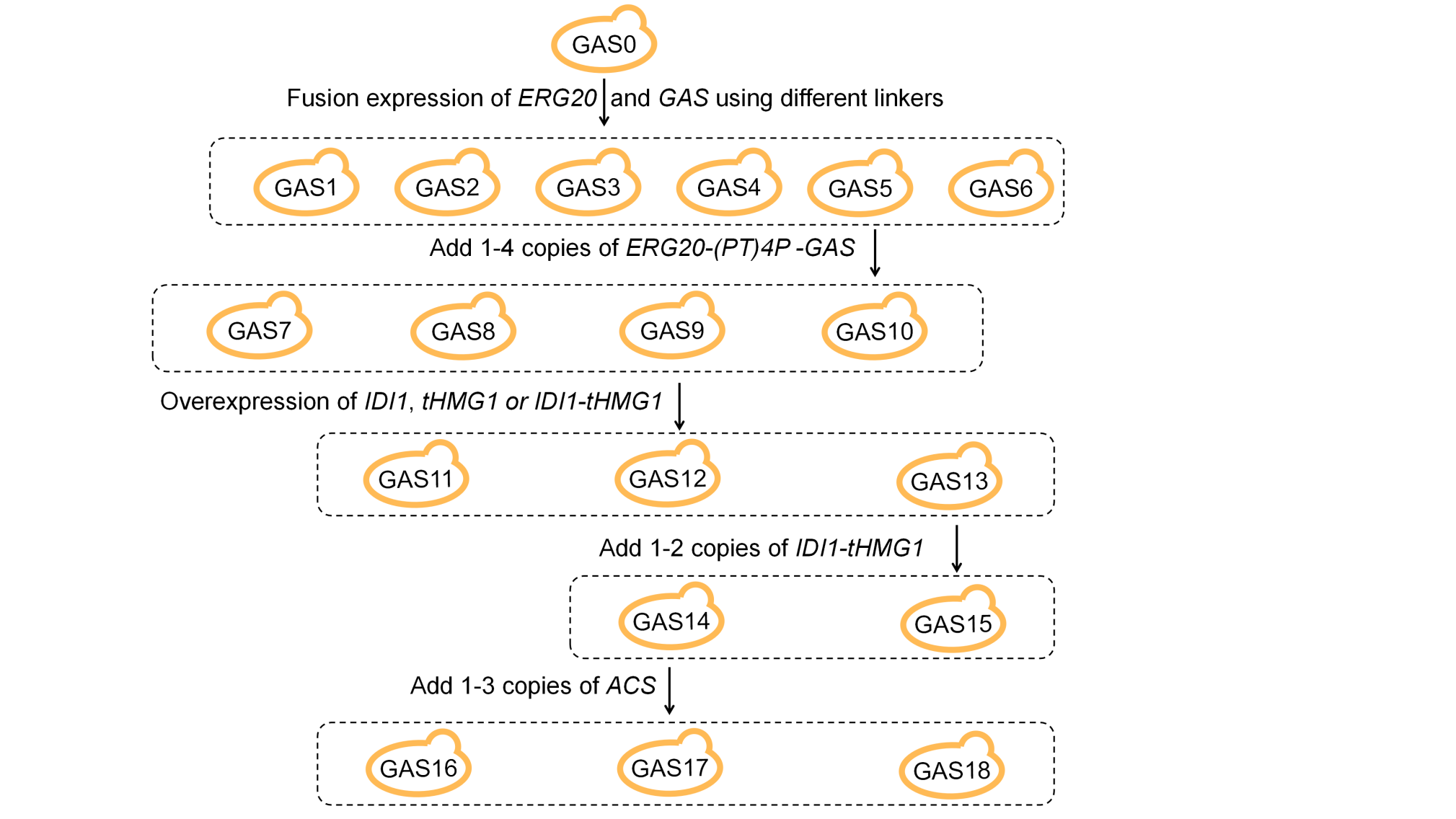


**Supplementary Fig. S1. Construction procedures of the engineered *P. pastoris* strains.**


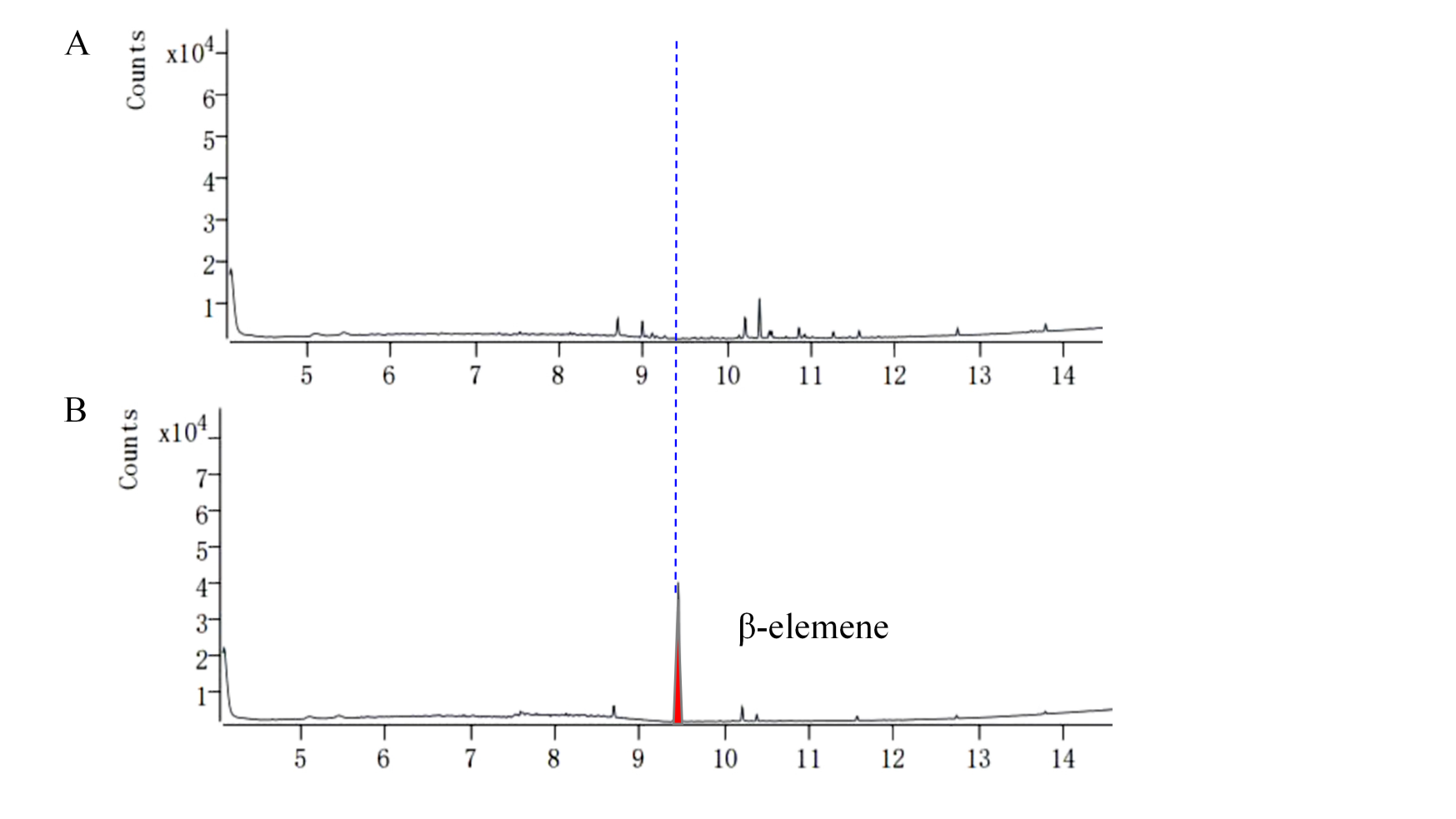


**Supplementary Fig. S2.** **GC-MS analysis of the production of β-elemene.** Compared with the control strain GAS0, the fermentation product of GAS1 showed a new peak at 9.4 min, whose MS spectrum was provided in Supplementary Figure S3.


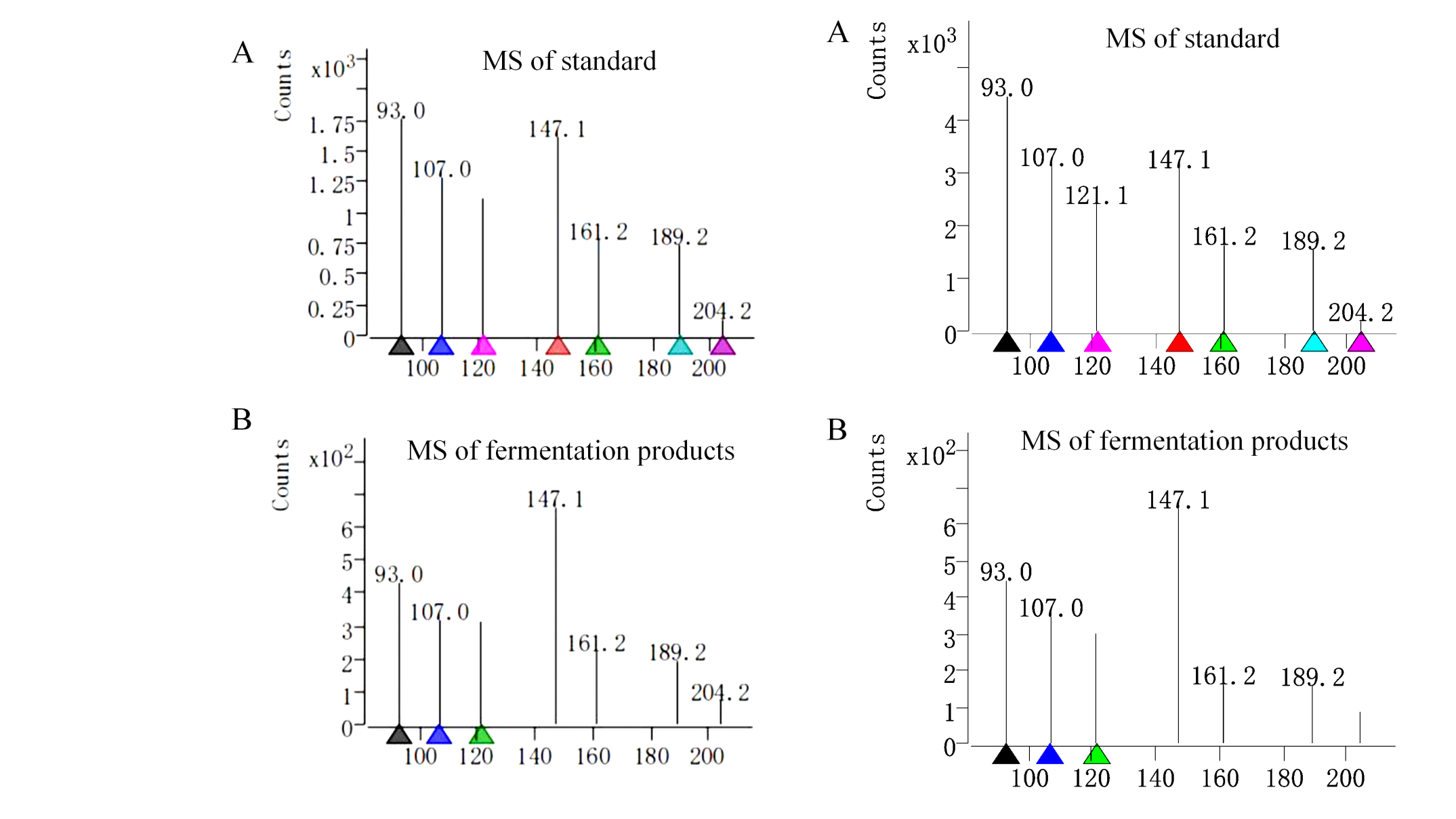


**Supplementary Fig. S3.** MS spectrum of β-elemene. The new peak at 9.4 min of the fermentation product (Supplementary Figure S2) was subject to MS analysis. By comparing with the MS fingerprint of the standard chemical, the new compound was determined to be β-elemene.


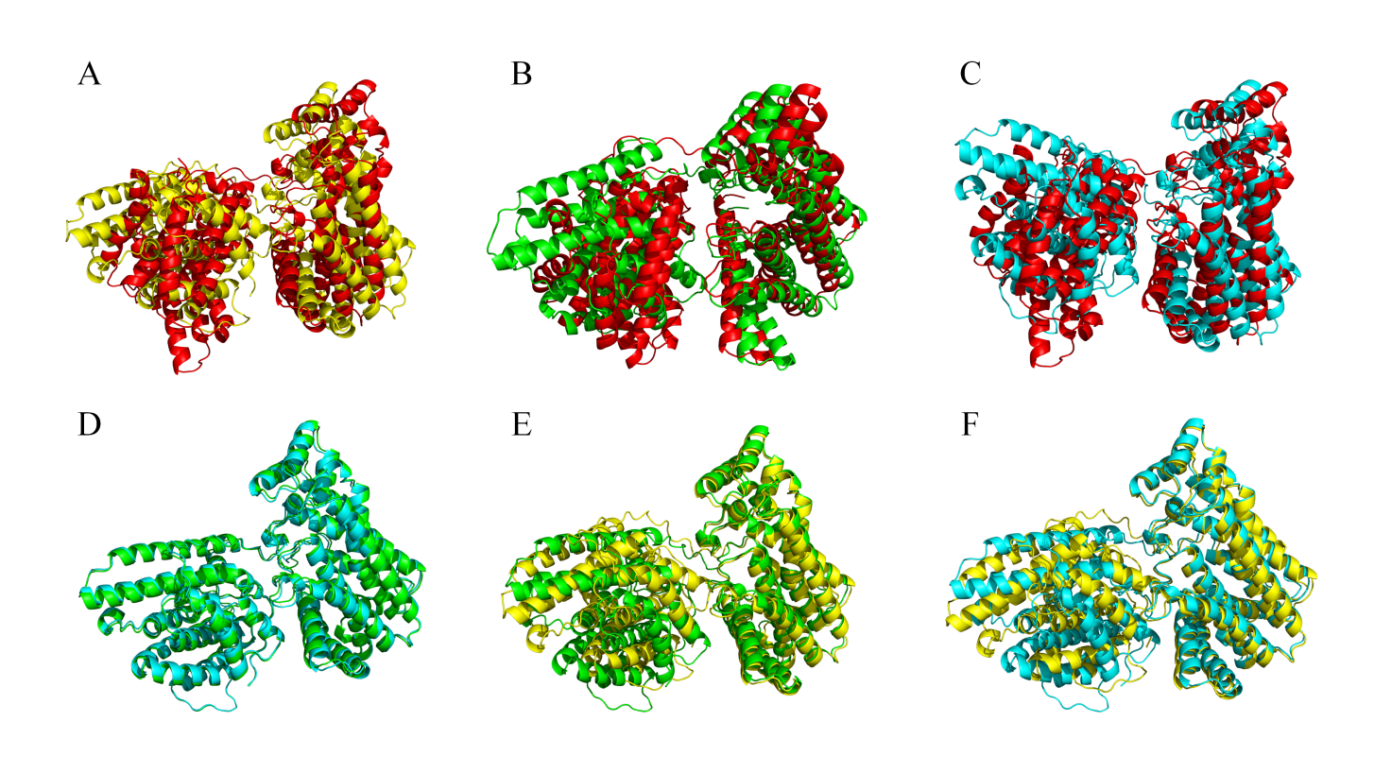


**Supplementary Fig. S4.** Comparison of spatial distances of two proteins (ERG20 and GAS) using different linkers. 3D homology modeling for ERG20 and GAS was constructed using the Alpha Fold2, which was illustrated as ribbon diagrams using PyMOL. Red represents for ERG20-(PT)4P-GAS, yellow represents for ERG20- (PA)5-GAS, green represents for ERG20-GGGGS-GAS, and cyan blue represents for ERG20-GGS-GAS, respectively.

**Supplementary Tables**

**Supplementary Table S1. A list of plasmids constructed in this study.**

| Name | Description | Reference |
| --- | --- | --- |
| HZP-gRNA-Int1 | PARS1, Zeo, *SERp*-Int1SgRNA^a^ | [(Gao J et al. 2022)](#_ENREF_1) |
| HZP-gRNA-Int12 | PARS1, Zeo, *SERp*-Int12SgRNA |  |
| HZP-gRNA-Int32 | PARS1, Zeo, *SERp*-Int32SgRNA |  |
| HZP-gRNA-Int33 | PARS1, Zeo, *SERp*-Int33SgRNA |  |
| HZP-gRNA-Int34 | PARS1, Zeo, *SERp*-Int34SgRNA |  |
| HGP-gRNA-Int20 | PARS1, G418, *SERp*-Int20SgRNA^a^ |  |
| HHP-gRNA-Int21 | PARS1, HygB, *SERp*-Int21SgRNA^a^ |  |
| HZP-gRNA-Int31 | PARS1, Zeo, *SERp*-Int31SgRNA |  |
| HZP-gRNA-Int11 | PARS1, Zeo, *SERp*-Int11SgRNA |  |
| HZP-gRNA-Int16 | PARS1, Zeo, *SERp*-Int16SgRNA |  |
| HZP-gRNA-Int6 | PARS1, Zeo, *SERp*-Int6SgRNA |  |
| Int1-TEF1 | Int1-HAup-*TEF1p-BsaI*-*AOX1t*-HAdown^b^ | This study |
| Int12-TEF1 | Int12-HAup-*TEF1p-BsaI*-*AOX1t*-HAdown | This study |
| Int32-TEF1-GAP | Int32-HAup-*TEF1p-BsaI*-*AOX1t*-*GAPp-AtaII-CYC1t*-HAdown | This study |
| Int33-TEF1-GAP | Int33-HAup-*-TEF1p-BsaI-AOX1t-GAPp-AtaII-CYC1t-*-HAdown | This study |
| Int34-TEF1-GAP | Int34-HAup-*TEF1p-BsaI-AOX1t-GAPp-AtaII-CYC1t-*HAdown | This study |
| Int20-TEF1 | Int20-HAup-*TEF1p-BsaI*-*AOX1t*-HAdown | This study |
| Int21-TEF1 | Int21-HAup-*TEF1p-BsaI*-*AOX1t*-HAdown | This study |
| Int31-TEF1 | Int31-HAup-*TEF1p-BsaI*-*AOX1t*-HAdown | This study |
| Int11-TEF1 | Int11-HAup-*TEF1p-BsaI*-*AOX1t*-HAdown | This study |
| Int16-TEF1 | Int16-HAup-*TEF1p-BsaI*-*AOX1t*-HAdown | This study |
| Int6-TEF1 | Int6-HAup-*TEF1p-BsaI*-*AOX1t*-HAdown | This study |
| Int1-TEF1-GAS | Int1-HAup-*TEF1p-GAS-AOX1t*-HAdown | This study |
| Int1-TEF1-ERG20 | Int1-HAup-*TEF1p-ERG20-AOX1t*-HAdown | This study |
| Int1-TEF1-ERG20-GAP-GAS | Int1-HAup-*TEF1p-ERG20-AOX1t-GAPp-GAS-CYC1t*-HAdown | This study |
| Int1-TEF1-ERG20-GGS-GAS | Int1-HAup-*TEF1p-ERG20-GGS-GAS-AOX1t*-HAdown | This study |
| Int1-TEF1-ERG20-GGGGS-GAS | Int1-HAup-*TEF1p-ERG20-GGGGS-GAS-AOX1t*-HAdown | This study |
| Int1-TEF1-ERG20-(PT)4P-GAS | Int1-HAup-*TEF1p-ERG20-(PT)4P-GAS-AOX1t*-HAdown | This study |
| Int1-TEF1-ERG20-(PA)5-GAS | Int1-HAup-*TEF1p-ERG20-(PA)5-GAS-AOX1t*-HAdown | This study |
| Int12-TEF1-ERG20-(PT)4P-GAS | Int12-HAup-*TEF1p-ERG20-(PT)4P-GAS-AOX1t*-HAdown | This study |
| Int32-ERG20-(PT)4P-GAS-2copy | Int32-HAup-*TEF1p-ERG20-(PT)4P*-*AOX1t*-*GAPp-* *ERG20-(PT)4P-GAS-CYC1t*-HAdown | This study |
| Int33-ERG20-(PT)4P-GAS-2copy | Int33-HAup-*TEF1p-ERG20-(PT)4P*-*AOX1t*-*GAPp-* *ERG20-(PT)4P-GAS-CYC1t*-HAdown | This study |
| Int34-ERG20-(PT)4P-GAS-2copy | Int34-HAup-*TEF1p-ERG20-(PT)4P*-*AOX1t*-*GAP-* *ERG20-(PT)4P-GAS-CYC1t*-HAdown | This study |
| Int20-TEF1-IDI1 | Int20-HAup-*TEF1p-IDI1*-*AOX1t*-HAdown | This study |
| Int20-TEF1-tHMG1 | Int20-HAup-*TEF1p-tHMG1*-*AOX1t*-HAdown | This study |
| Int20-TEF1-IDI1-tHMGR1 | Int20-HAup-*TEF1p-IDI1*-*AOX1t*-*GAPp-tHMG1-CYC1t*-HAdown | This study |
| Int21-TEF1-IDI1-tHMGR1 | Int21-HAup-*TEF1p-IDI1*-*AOX1t*-*GAPp-tHMG1-CYC1t-*HAdown | This study |
| Int31-TEF1-IDI1-tHMGR1 | Int35-HAup-*TEF1p-BsaI-AOX1t-GAPp-AtaII-CYC1t-*HAdown | This study |
| Int11-TEF1-ACS | Int11-HAup-*TEF1p-ACS*-*AOX1t*-HAdown^c^ | This study |
| Int16-TEF1-ACS | Int16-HAup-*TEF1p-ACS*-*AOX1t*-HAdown | This study |
| Int6-TEF1-ACS | Int31-HAup-*TEF1p-ACS*-*AOX1t*-HAdown | This study |

Note:

^a^ Zeo, Zeocin; HygB, Hygromycin B; G418, Geneticin; Amp, Ampicillin.

^b^ HAup and HAdown represent the upstream (~500 bp) and downstream (~500 bp) homology arm sequences.

^c^*ACS* represents the feedback inhibition insensitive acetyl-CoA synthetase mutant (*ACS^L641P^*) from *Salmonella enterica*, which has been codon-optimized for expression in *S. cerevisiae* (Lian J et al. 2014).

**Supplementary Table S2. A list of primers used in this study.**

| Oligo | Sequences (5’-3’) |
| --- | --- |
| GAS-F | cattttagttattcgccaacatggaaaaatttacgttccctaacttg |
| GAS-R | catcctcttgattagaatctagtcagctcgcaagaagctctaac |
| ERG20-F | cactacatacattttagttattcgccaacatgtccaaagaagtagcagctaag |
| ERG20-R | ctgacatcctcttgattagaatctagttatttggttctcttgtagatcttgtcaaag |
| ERG20-link1-GAS-F | caagatctacaagagaaccaaaggcggcagcatggaaaaatttacgttccctaacttg |
| ERG20-link1-GAS-R | catcctcttgattagaatctagtcagctcgcaagaagctctaac |
| ERG20-link2-GAS-F | agatctacaagagaaccaaaggaggtggtggatctatggaaaaatttacgttccctaacttg |
| ERG20-link2-GAS-R | catcctcttgattagaatctagtcagctcgcaagaagctctaac |
| ERG20-link3-GAS-F | agatctacaagagaaccaaacctactccaactccaacacctacacctatggaaaaatttacgttccctaacttg |
| ERG20-link3-GAS-R | catcctcttgattagaatctagtcagctcgcaagaagctctaac |
| ERG20-link4-GAS-F | agatctacaagagaaccaaaccagctcctgctccagctcctgcaatggaaaaatttacgttccctaacttg |
| ERG20-link4-GAS-R | catcctcttgattagaatctagtcagctcgcaagaagctctaac |
| IDI1-F | ggacgcaactatcagacgaaaatac |
| IDI1-R | aaacgcactgaaaatcggcactacttacataaaacccaaggagatttcagaggagttgg |
| tHMG1-F | gaccagatctcagagtatgtgtgctcaaactagaatagttctgctactctggtcccaag |
| tHMG1-R | ggagagagttcccgctagaac |
| ACS-F | cattttagttattcgccaacatgtcacaaacacacaaacatg |
| ACS-R | tcctcttgattagaatctagtcatgatggcatagcaatagcttg |
| Int1-donor-F | ctgggcagtagtgaattggttg |
| Int1-donor-R | acattgttcgtgaggctaatcc |
| Int12-donor-F | ctgggcagtagtgaattggttg |
| Int12-donor-R | acattgttcgtgaggctaatcc |
| Int32-donor-F | gactggtgccttgattttcg |
| Int32-donor-R | tcgtaagaagggtctgtgatag |
| Int33-donor-F | aaagagagagtctaaaagtggtg |
| Int33-donor-R | attataattagcacggtgttgc |
| Int34-donor-F | tcggaatcctgtgtctggaag |
| Int34-donor-R | aattataagacaggactgttgtgg |
| Int20-donor-F | ccgatcattgcatagataccaaag |
| Int20-donor-R | ggtcagaattacttcacacttc |
| Int21-donor-F | gatctttgatcagattcacgtttag |
| Int21-donor-R | accatgaaactgccattgaatac |
| Int31-donor-F | gtgttctacgctagaaactcc |
| Int31-donor-R | tacgtgttagcaaagttgacg |
| Int11-donor-F | gtatgcaaacatttcgtcctcc |
| Int11-donor-R | tggtaaatggtgaagttggc |
| Int16-donor-F | acaccaccacaaatacctaccaatg |
| Int16-donor-R | acctgggagaatgttttcaatctgg |
| Int6-donor-F | agtaagggcagtgtaccatag |
| Int6-donor-R | acattaagaaccaagatcatgca |

**Supplementary Table S3. A list of gene coding sequences used in this study.**

| Gene | Coding sequences (5’-3’) |
| --- | --- |
| *GAS* | atggaaaaatttacgttccctaacttgtattgtccctttccggaacggaaaaatccatacagcgagtttcttcaggactatgctttgcagtgggtgatcagatttaagcttatcgattccgaaagcctgtaccagaggttcagtaaggccaagttctatttgctaaccgccggggcctatcctcattgtcaactcgaggaactcaagatcgcaaatgacgtgatttcatggttatttatctgggacgaccaatgtgatatttcagacctaggaaagaaacccgaactcctaaagacttggtgcaatcggtttttagaaattcttaacggcgcagagttaacgcctgatgatctcccactgggatttgcattaagagacataaggaatcgaattataaatcgagggggtattaccttcttccaccacttcgtccgaaactttgaagattattttcacggttgtattgaggaggctcataatagagttaatgtttctgtcccggatgttgaggcttacataaaaatcaggagtgcgaatgcagccgcagccctatgcctgaacctgattgagttctgcgatcgcgtgatgattccctactcgctccgcaatcatgagaccttgaagaaacttactcaaatgacgataaacatcttagcatggtccaacgatatatttagtgcgccacgtgaaatagctaacggcgaagtacataatctagtattcgttatccaccaccatcaaaaaatcccgctggagaaagcgatgctagcagctgcggcgatgcacaaccatgaagtacagaaattggtcaacctcgagtccaaaatagcctcattctcggcggagacagacgctgaaattacaaagtatatatcggggttgcatgcttggatacgtggaaacctggactggtacgcccactctggccgctaccaaatcacagaaaagttagagcttcttgcgagctga |
| *ERG20* | atgtccaaagaagtagcagctaagaacaaagtgaagttccttgcgttctttccagacctcgtgtttgagctggaagtcatgctggaaaattacggcatgcctgctgatgcaatcaactggttcaagagatccttgaactacaacactcctggtggaaaactcaacagaggtatctctgttgtcgacacctatgtcattctcaaggggtacaagtcatgggacgagttaagtgccgaagagttcaagaaagctgctattttaggatggtgtgtcgaattgctacaagcctacttcctggtcgctgatgatatgatggatcagtcaatcaccagaagaggccaaccttgttggtacaaggttgaaaatgtaggaaacattgccattaacgactctttcatgctagaaggtgctatctataaaattttacgcaagtacttcaagaaagaatcttattatgctgacttgttggatctgctccatgaggtaactttccaaactgagttaggccaattgttggatttgatcaccgctccagaagaccacgtcgatctttccaagtttaccccagccaagcattcttttattgtcatttttaaaactgcttattactcgttctacctgccagttgtgatggcaatgtacctgagcggtattacccatgaaaaagatctacaacaggctgagcacatcttgattccgctaggtgaatacttccagattcaagatgactaccttgattgttttggtaaaccagaagacattggaaagatcggaactgacatccaggataataagtgttcttgggtcatcaaccaagctttgaggttggctagccccgaacagagacagatattggatgaaaactatggacgcaaagatgccgacaaggaagccaaatgtaaagaagttttcgaccagctcgacattgccgggaagtacaaggcttatgaggagaacatcggcaaagaattgcagaagagaatcgctgacacagaagaagacaggggattcaagaaggaagtcttccaagttttctttgacaagatctacaagagaaccaaataa |
| *tHMG1* | atggctgcagaccaattggtgaaaactgaagtcaccaagaagtcttttactgctcctgtacaaaaggcttctacaccagttttaaccaataaaacagtcatttctggatcgaaagtcaaaagtttatcatctgcgcaatcgagctcatcaggaccttcatcatctagtgaggaagatgattcccgcgatattgaaagcttggataagaaaatacgtcctttagaagaattagaagcattattaagtagtggaaatacaaaacaattgaagaacaaagaggtcgctgccttggttattcacggtaagttacctttgtacgctttggagaaaaaattaggtgatactacgagagcggttgcggtacgtaggaaggctctttcaattttggcagaagctcctgtattagcatctgatcgtttaccatataaaaattatgactacgaccgcgtatttggcgcttgttgtgaaaatgttataggttacatgcctttgcccgttggtgttataggccccttggttatcgatggtacatcttatcatataccaatggcaactacagagggttgtttggtagcttctgccatgcgtggctgtaaggcaatcaatgctggcggtggtgcaacaactgttttaactaaggatggtatgacaagaggcccagtagtccgtttcccaactttgaaaagatctggtgcctgtaagatatggttagactcagaagagggacaaaacgcaattaaaaaagcttttaactctacatcaagatttgcacgtctgcaacatattcaaacttgtctagcaggagatttactcttcatgagatttagaacaactactggtgacgcaatgggtatgaatatgatttctaaaggtgtcgaatactcattaaagcaaatggtagaagagtatggctgggaagatatggaggttgtctccgtttctggtaactactgtaccgacaaaaaaccagctgccatcaactggatcgaaggtcgtggtaagagtgtcgtcgcagaagctactattcctggtgatgttgtcagaaaagtgttaaaaagtgatgtttccgcattggttgagttgaacattgctaagaatttggttggatctgcaatggctgggtctgttggtggatttaacgcacatgcagctaatttagtgacagctgttttcttggcattaggacaagatcctgcacaaaatgttgaaagttccaactgtataacattgatgaaagaagtggacggtgatttgagaatttccgtatccatgccatccatcgaagtaggtaccatcggtggtggtactgttctagaaccacaaggtgccatgttggacttattaggtgtaagaggcccgcatgctaccgctcctggtaccaacgcacgtcaattagcaagaatagttgcctgtgccgtcttggcaggtgaattatccttatgtgctgccctagcagccggccatttggttcaaagtcatatgacccacaacaggaaacctgctgaaccaacaaaacctaacaatttggacgccactgatataaatcgtttgaaagatgggtccgtcacctgcattaaatcctaa |
| *IDI1* | atgactgccgacaacaatagtatgccccatggtgcagtatctagttacgccaaattagtgcaaaaccaaacacctgaagacattttggaagagtttcctgaaattattccattacaacaaagacctaatacccgatctagtgagacgtcaaatgacgaaagcggagaaacatgtttttctggtcatgatgaggagcaaattaagttaatgaatgaaaattgtattgttttggattgggacgataatgctattggtgccggtaccaagaaagtttgtcatttaatggaaaatattgaaaagggtttactacatcgtgcattctccgtctttattttcaatgaacaaggtgaattacttttacaacaaagagccactgaaaaaataactttccctgatctttggactaacacatgctgctctcatccactatgtattgatgacgaattaggtttgaagggtaagctagacgataagattaagggcgctattactgcggcggtgagaaaactagatcatgaattaggtattccagaagatgaaactaagacaaggggtaagtttcactttttaaacagaatccattacatggcaccaagcaatgaaccatggggtgaacatgaaattgattacatcctattttataagatcaacgctaaagaaaacttgactgtcaacccaaacgtcaatgaagttagagacttcaaatgggtttcaccaaatgatttgaaaactatgtttgctgacccaagttacaagtttacgccttggtttaagattatttgcgagaattacttattcaactggtgggagcaattagatgacctttctgaagtggaaaatgacaggcaaattcatagaatgctataa |
| *ACS** | atgtcacaaacacacaaacatgctattcctgcgaatatcgctgacaggtgcttaatcaaccctgaacaatacgaaacgaagtacaagcagtctatcaacgatcctgatactttctggggcgagcaaggtaagatactcgattggattactccatatcaaaaggtcaaaaacacatcctttgctcctggaaatgtgtcaatcaagtggtacgaggacggcactctaaacctagctgctaattgcttggatcgacacctccaggaaaatggtgacagaacggcaatcatttgggaaggtgatgatacttctcaatctaagcacatctcctacagagagttacacagagatgtttgcagattcgcgaatactttactggacctgggtatcaaaaagggcgatgttgtggcaatctacatgcctatggtcccagaggcagctgtggcaatgttggcctgtgccagaataggagcagtccatagcgttatctttggcggattctcccctgaagccgttgctgggagaatcattgactcatcaagtagattagttatcactgccgacgaaggtgttagagcaggtagatccatcccattgaagaaaaacgttgatgacgcgttgaaaaacccaaacgttacgagtgtggagcatgtaattgtactaaagcgtaccggctctgatatagactggcaggaaggtagggatttgtggtggagagatcttattgagaaagcaagtccagaacaccaaccagaagcaatgaatgcggaagatccattgttcatcttgtatacatctgggtcaactggcaaaccaaaaggtgttttgcatacaacaggtggttatctcgtatacgccgcaacaacctttaagtacgtttttgattaccatccaggtgatatctactggtgtaccgctgatgtcggttgggttactggtcatagttacctgctttacggtccactggcatgcggcgcaaccactttgatgtttgaaggagtaccaaactggccaaccccagccaggatgtgtcaagtggtcgataaacaccaagtgaacatattgtacacagccccaaccgccattagagcgctaatggccgaaggagataaggcgattgagggaacagatagaagtagcctacgtatcttaggatccgttggcgagccaatcaatccagaagcttgggaatggtattggaaaaagattggtaaggaaaagtgtccagtagtggatacatggtggcaaactgaaacaggtggattcatgattacacctcttccaggtgcaatagaattgaaggctgggtctgctactaggcctttcttcggcgtccaacctgctttagtagacaacgaagggcatccacaagagggggcaacagaaggcaatctagtgataactgattcctggcctggtcaggctagaacattgtttggtgatcacgaaagattcgaacaaacctatttctcaactttcaaaaacatgtatttcagcggtgacggtgcgagaagagacgaagatgggtactactggattaccggcagagtagatgacgtccttaacgtatctggacatcgtctgggtacagctgagattgagtcagctttagttgctcatcctaagattgctgaagctgcagtcgttggcatcccacacgctatcaagggtcaagccatatacgcatatgttacactcaaccatggtgaggaaccatctccagagctatacgcagaggtcagaaattgggttcgaaaggaaatagggcctttagccacaccagatgttttgcattggacagattcattgcctaagacaagatctggaaagattatgagacgtatacttagaaagatcgccgccggagatacgtctaacttaggtgatacttctactcttgccgatccaggcgtggtcgaaaaacctttagaggaaaaacaagctattgctatgccatcatga |

**Supplementary Table S4. A list of integration sites used in this study.**

|  | Upstream ORF | Downstream ORF |
| --- | --- | --- |
| Int1 | PAS_FragB_0066 | PAS_FragB_0067 |
| Int6 | PAS_chr2-1_0010 | PAS_chr2-1_0011 |
| Int11 | PAS_chr3_0154 | PAS_chr3_0156 |
| Int12 | PAS_chr3_1202 | PAS_chr3_0618 |
| Int16 | PAS_chr3_1065 | PAS_chr3_1067 |
| Int20 | PAS_chr4_0465 | PAS_chr4_0467 |
| Int21 | PAS_chr4_0576 | PAS_chr4_0575 |
| Int31 | PAS_chr2-1_0727 | PAS_chr2-1_0728 |
| Int32 | PAS_chr2-2_0142 | PAS_chr2-2_0143 |
| Int33 | PAS_chr1-1_0053 | PAS_chr1-1_0054 |
| Int34 | PAS_chr3_0053 | PAS_chr3_0054 |

**Supplementary Table S5. A list of yeast extract and peptone used in this study.**

| Yeast extract | Description | Order NO. | Vendor and order link |
| --- | --- | --- | --- |
| Y | Yeast extract. It provides a variety of nutrients for microorganisms. | A515245 | Sangon Biotech, https://www.sangon.com/productDetail?productInfo.code=A515245 |
| Y1 | Bacto^TM^ yeast extract. | BD212750 | ThermoFisher SCIENTIFIC, www.thermofisher.cn/order/catalog/product/212750 |
| Peptone | Description | Order NO. | Vendor and order link |
| P | Peptone, pepsin digest of blood fiber-based proteins. Routinely used as medium component for microbial growth | A505247 | Sangon Biotech, www.sangon.com/productDetail?productInfo.code=A505247 |
| P1 | Bacto^TM^ peptone, enzymatic digest of bovine and porcine animal proteins | BD211677 | ThermoFisher SCIENTIFIC, www.thermofisher.cn/order/catalog/product/211677 |
| P2 | Peptone A (Meat peptone from Bovine) | A610213 | Sangon Biotech, www.sangon.com/productDetail?productInfo.code=A610213&jump_from=1_05_37_01 |

**Supplementary References**

Gao J, Xu J, Zuo Y et al (2022) Synthetic biology toolkit for marker-less integration of multigene pathways into *Pichia pastoris* via CRISPR/Cas9. ACS Synth Biol 11:623-633

Lian J, Si T, Nair. N et al (2014) Design and construction of acetyl-CoA overproducing *Saccharomyces cerevisiae* strains. Metab Eng 24:139-149
